# Sense of Agency, Affectivity and Social-Ecological Degradation: An Enactive and Phenomenological Approach

**DOI:** 10.3389/fpsyg.2022.911092

**Published:** 2022-07-07

**Authors:** Jesús M. Siqueiros-García, David Manuel-Navarrete, Hallie Eakin, Laura Mojica, Lakshmi Charli-Joseph, Patricia Pérez-Belmont, Beatriz Ruizpalacios

**Affiliations:** ^1^Unidad Académica del Instituto de Investigaciones en Matemáticas Aplicadas y en Sistemas (IIMAS) en Yucatán, Institute of Applied Mathematics and Systems Research, Universidad Nacional Autónoma de México, Mexico City, Mexico; ^2^School of Sustainability, Arizona State University, Tempe, AZ, United States; ^3^División de Ciencias Sociales y Humanidades, Universidad Autónoma Metropolitana Unidad Cuajimalpa, Mexico City, Mexico; ^4^Embodied Cognitive Science Unit, Okinawa Institute of Science and Technology, Okinawa, Japan; ^5^Laboratorio Nacional de Ciencias de la Sostenibilidad, Instituto de Ecología, Universidad Nacional Autónoma de México, Mexico City, Mexico; ^6^Posgrado en Ciencias de la Sostenibilidad, Universidad Nacional Autónoma de México, Mexico City, Mexico

**Keywords:** affectivity, social-ecological degradation, agency, form of life, phenomenology, affordances, enactivism

## Abstract

In the last few years, there has been an interest in understanding the impact of environmental change and degradation on people's affective life. This issue has become particularly pressing for populations whose form of life is heavily dependent on ecosystem services and functions and whose opportunities for adaptation are limited. Based on our work with farmers from the Xochimilco urban wetland in the southwest of Mexico City, we begin to draw a theoretical approach to address and explain how environmental degradation impacts people's affective life and sense of agency. Farmers who were part of our project referred to a sense of despair and helplessness toward the loss of the ecosystem and their traditional farming-based form of life. From the perspective of phenomenology, enactivism and ecological psychology, we argue that the loss of this form of life in the area is related to the degradation of socio-ecological systems, limiting the opportunities for people to relate meaningfully to others and the environment. We posit that losing meaningful interaction with the environment generates a feeling of loss of control while leading farmers to feel frustrated, anxious and stressed. Such affective conditions have a direct impact on their sense of agency. In terms of adaptation, the negative interaction between degradation, affective states and a diminished sense of agency can create a downward spiral of vulnerability, including political vulnerability.

## Introduction

Since Albrecht's article on solastalgia, there has been growing interest in understanding the relationship between ecosystems and human affective states (Albrecht, 2011, 2005, 2019; Galway et al., 2019). Solastalgia is a neologism coined by Albrecht to refer to the loss of solace, the grievance and lasting desolation resulting from the loss of one's home and territory due to environmental change (Albrecht, 2005, 2019). Ecological degradation has been shown to manifest as harm to people's emotional life and affectivity, with solastalgia, anxiety and depression being the most common conditions (Galway et al., 2019; Stanley et al., 2021).

Much of the research on affective health, wellbeing, ecological degradation and climate change has been done in the US or Australia while many other parts of the world are underrepresented in these studies (Tschakert et al., 2013; Galway et al., 2019). This matters because populations with livelihoods directly dependent on ecosystem services and functions are found all over the world. These populations tend to be the most vulnerable to solastalgia as their immediate needs and livelihoods are directly affected by environmental degradation (Tschakert et al., 2013; Poma, 2018; Iniguez-Gallardo et al., 2021).

Moreover, it has been acknowledged that affectivity, emotions and mental health needs to be addressed from a perspective that deeply understands their embodied nature. Such understanding has been clearly expressed by Feminist Political Ecologists and Emotional Geographers, who have been claiming for some time the recognition of the relevance of the body as the locus of experience, for example, in relation to climate change, power, inequalities, gender, subjectivity, etc. (Bondi, 2005; Pile, 2010; Elmhirst, 2015, 2011; Sultana, 2015; Bergmann et al., 2020; González-Hidalgo and Zografos, 2020; Nightingale et al., 2022).

Building on previous work (Eakin et al., 2019), this article theoretically elaborates the connection between individuals' affective life, sense of agency, and the ecosystems to which they are inextricably interrelated and dynamically coupled. We develop a phenomenologically inspired approach to theorize affective stress due to environmental degradations, in which we assume that the erosion of a social-ecological system^1^ is the erosion of a form of life that erases the distinction of the physical, the ecological and the different social domains of action (social, cultural, economic, and political) (Ingold, 2004; Linton and Budds, 2014; Bergmann et al., 2020). In this sense, we posit that environmental anguish and its psychological toll cannot be exclusively attributed to the disappearance of vegetation and animals, or even of a landscape. Instead, we show that environmental anguish results from the erosion of a social-ecological system that makes possible the permanence of the ways and practices of a community, given that ultimately is the social-ecological system what sustains people's deepest and shared know-how.

The content of this manuscript comes from two and a half years of experimenting with Transformation-Laboratories (T-labs) in the urban wetland of Xochimilco in the southwest of Mexico City (Charli-Joseph et al., 2018; Eakin et al., 2021). The T-Lab project was a participatory process that created an adaptive space for experimentation with local residents and farmers, academics, NGOs and government officials. The project focused on exploring diverse activities and tools that could promote a sense of agency among participants, individually and as a group (Adger, 2003; Aldrich and Meyer, 2015). We were specifically focused on those whose lives are negatively affected by the degradation of Xochimilco wetlands, a social-ecological system that has historically supported place-based meanings and livelihoods grounded in tradition and shared values.

The impact of environmental degradation on people's affective life and emotions is particularly salient in understanding people's response to climate change. Such efforts go beyond understanding changes in moods and emotions to include what Colombetti refers to it as *primordial affectivity*. In her words: “Affectivity […] refers broadly to a *lack of indifference*, and rather a *sensibility* or *interest* for one's existence” (Colombetti, 2014, p. 1). Primordial affectivity suggests a deeper connection between affectivity and sense of agency.

The relation between affectivity and sense of agency has been described in studies about depression, anxiety and other similar conditions (Ratcliffe, 2008; Slaby, 2012; Fuchs and Koch, 2014). In the context of adaptation to climate change, psychological stress may lead to a diminished sense of agency, reducing motivation and the capacity to seize opportunities and identify leverage points for action. In the worst-case scenario, populations may get trapped in a vicious circle where environmental degradation reduces people's sense of agency, increasing psychological stress and leading to inaction, and further environmental degradation.

The next section presents a diagnosis of Xochimilco's social-ecological degradation and the conditions that give rise to anxiety, stress and solastalgia among Xochimilco's farmers (known as chinamperos). Section Exploring Agency Through a T-Lab describes briefly the T-Lab of Xochimilco and results. Section Agency introduces a theoretical framework on agency based on different, but highly related, traditions of phenomenology and embodied cognition research, particularly enactivism and ecological psychology. We argue that combining these traditions allows interpreting the complex connections between the material degradation of life and the subjective emotions of farmers vis-à-vis political processes and power relations. In Section The Collective Nature and Politics of Affectivity we build on our T-lab experiments to explore the collective nature and politics of affectivity.

## Xochimilco Social-Ecological Wetland

Xochimilco is an urban wetland in Mexico City that has been inhabited for more than 1,000 years. The wetland is one of the last remaining waterbodies of what was once five shallow lakes that covered the Mexico City inland basin. The people of Xochimilco were living, dying, dwelling and farming on this land at least 400 years before the Aztecs founded Tenochtitlan—the pre-Columbian city where Mexico City is located now—in 1325. In this section, we briefly describe chinampa farming, a unique type of social-ecological system in Xochimilco, the historical process of its deterioration and how this has been affecting traditional farming practices, i.e., the shared practical know-how of the community. Our identification of the chinampas as a social-ecological system is intentional: we conceive of the chinampas as a unit formed as a dynamic system of interconnections and dependencies of material transformations and processes resulting from farming practices.

Xochimilco's chinampas are raised beds (or artificial islands) built inside the lake with sediment (Armillas, 1971; Rojas, 1995). This farming system provided an abundance of corn and many other goods to the area in pre-Hispanic times. The chinampas are surrounded by wide waterways, used for motorized and manual punt transportation, and small narrow channels, or *apantles*, that allow natural irrigation. The soil of the raised beds is contained by reeds and the native species *Ahuejote*, a willow tree (Armillas, 1971). These farming practices are known as *chinamper*í*a*, and they are recognized internationally as part of the world's cultural heritage.

Today this once highly productive system is endangered from significant degradation of the wetland ecosystem. As is the case in almost anywhere in the world, there is no single reason behind Xochimilco's environmental decline, and as almost anywhere, it is not only the biophysical component that is under assault but also the whole social and cultural fabric that was tightly knitted over centuries of social-ecological history. It should be added that Xochimilco is a RAMSAR site^2^ and UNESCO heritage site.^3^ As we demonstrate, it is the degradation of this system that has led to the current situation of a sense of loss of agency among farmers.

Degradation can be understood as changes that negatively impact on the social and environmental life support systems; degradation can lead a system to cross a tipping point such that life becomes unsustainable. Such changes can have many origins. Some changes are sociotechnical in nature, such as the intensive use of excess of synthetic nitrogen and phosphorous fertilizers. Others are geological, such as the earthquake that fractured the chinampas in 2017. And some others are political and economic in origin, relating to a lack of access to economic resources or political support.

More specifically, we understand the degradation of Xochimilco and the chinampas social-ecological system as rooted in four driving forces: (a) Mexico City's water demand, (b) uncontrolled urbanization of the area, (c) farming policies, and (d) the market to which farmers are subjected. We address each of these drivers below.

### Mexico City's Water Demand

While the water of Xochimilco has supplied for populations in the Mexico City basin since pre-Hispanic times (Tellman et al., 2018), the real impact of satisfying this demand began in the early 1900s when the Mexican president, Porfirio Díaz ordered the construction of an aqueduct to transport water from Xochimilco to the City. This particular event is seen as one of many hallmarks in the decline of the chinampería (Jiménez et al., 2020). The springs that fed the Xochimilco wetland supplied greater Mexico City until the 1950s when Xochimilco Lake was largely drained, significantly affecting the integrity of the chinampas (Tellman et al., 2018).

To restore the viability of the wetland's water channels, in 1959 the government of Mexico City fed the wetland with minimally treated wastewater, leading to the extinction of some local species due to the poor water quality. The drying up of the springs feeding the wetland and its channels increased the salinity of chinampa soils, substantially limiting their productive capacity. The wetland continues to be maintained with wastewater to the present (Jiménez et al., 2020; Gómez Aíza et al., 2021). The social and economic impacts of this historical process led to a reduction of the farming population from 38.6% in 1960 to 3.1% in the 1980s (Jiménez et al., 2020, p. 32).

### Uncontrolled Urbanization

Urban growth is the other important factor deeply affecting the social and ecological dynamics of Xochimilco. After the Mexican revolution of 1910, the land in and around the wetland was under usufruct tenure (*ejidal*); however, overtime the land tenure situation has become increasingly ambiguous, without well-defined property documentation. As a result, the land has been subjected to all forms of usage, trade, and exploitation, and has enabled both informal and illegal practices. From the 1960s until 2000, Xochimilco experienced a constant influx of people looking for a better life in the city, generating annual population growth rates of 5%. This compared to 2.5% for the rest of the City in the same period. Between 2000 and 2012, Xochilmico's population increased at 12.07% while Mexico City grew to a 2.85% rate (Ezcurra, 1990; González Pozo, 2016; Jiménez et al., 2020). This trend caused an unprecedented demand for housing, which neither Xochimilco nor the government of Mexico City was prepared to meet. As a result, the number of irregular human settlements has steadily increased, transforming land that once was for farming into an urban landscape. In this context, agriculture is also disincentivised by the alternative economic opportunities that have emerged with urbanization.

The implications of urban growth are immense for the chinampas and chinampería. Historically under traditional practices, the chinampas were not irrigated because they absorbed the required water from the channels that surrounded them. Those channels are now being filled, covered and transformed into roads and streets. The irregular human settlements that have expanded over the former chinampas lack sewage infrastructure; wastewater is thus dumped into the remaining channels, negatively affecting the water quality, and increasing the amount of organic residues and other pollutants generated by domestic activities (Mazari-Hiriart et al., 2008; Aguilar and Santos, 2011).

### Farming Policies

Inspired by the promise of Green Revolution technology, the Mexican government has incentivized intensive, monoculture farming, the use of agrochemicals and pesticides, as well as the use of greenhouses for ornamental plants. Among other practices farmers have substituted plastic seedling trays in place of the traditional mudflats used for sprouting seeds known as *chap*í*n* in the hope of increasing land productivity and profit. These policies were designed nationally without considering the local context of production. Farmers either became beneficiaries of such policies, or were excluded from them, leading to significant impacts on their collective sense of identity. In Mexico and elsewhere, these policies have been shown to impact famers' *narrative self* : their subjectivity evolves to embrace the political discourse, or they find themselves resisting the policy intent in the pursuit of alternative strategies (Bausch et al., 2015; Bausch, 2017, p. 50). Currently, green revolution practices are dominant, relegating traditional practices to a small number of chinamperos (Zambrano et al., 2020).

### Market Drivers

About 80% of the food consumed in Mexico City is produced in other states (Dieleman, 2017); hence, the city's food supply no longer depends on the chinampas production as was the case during Aztec and colonial times. Many of Xochimilco's remaining producers have now diversified into international exports of ornamental plants and flowers. Chinampa produce farmers mostly sell their products through intermediaries at the biggest wholesale food distribution center of Mexico City called *Central de Abastos*. To a lesser extent, some of them also sell in local markets, to restaurants or directly to consumers. Chinampa farmers compete with large scale producers from neighboring states that offer lower prices, reducing the profitability of chinampas, which is also affected by the biophysical issues of water and soil quality. According to some reports,^4^ chinampa farmers make a profit between USD 100 and 300 per month^5^; they own or lease between one and ten chinampas with an average area of 1,500 m^2^ (4,921.26 ft^2^), and the rents vary between USD 370 and 800 per year depending on the total area, water availability, and proximity to urban areas and roads. It is difficult for farmers to overcome these challenges of market prices and environmental instability, and that is why many of them seek other job opportunities to make a profit from other activities, which leads to land abandonment and in turn, favors urbanization.

Today Xochimilco is a heterogeneous mosaic of productivity, in which some chinampas have remained in production but many others have given into other uses. The drivers and outcomes we describe depict the systemic nature of degradation processes: the four driving forces identified act in an inter-dependent manner. Although its degradation is the result of historical processes, as in most complex systems, its effects are far from being random. It is a process in which events at one moment get entrenched and entangled and lead the way to future events in an unpredictable but systematic manner that can be intentionally disrupted (Manuel-Navarrete et al., 2019). This is where politics take place, and politics and power are co-constitutive of this complex system (Wise et al., 2014; Tellman et al., 2018).

Although further analysis would be required to identify the direct maneuvers of stakeholders and powerful groups in the politics of chinampería, it is clear from the trends described above are the result of distinct agendas and interests operating at different organizational and geographic scales, with serious implications for the power dynamics among chinampa farmers and other groups of interest such as political parties, NGOs and other organizations. The historical development of chinampería is not a neutral process; on the contrary, its political history is tangled with and contributes to the social-ecological dynamics experienced by producers: the high salinity of the earth, the prices of lettuce, the quality of the water in the channels. These processes are outcomes of negotiations among different stakeholders—e.g., farmers, agricultural brokers, political parties, among others—and opportunities for groups to exercise power and influence.

The degradation of the chinampa is far more than an issue of livelihood sustainability. Chinampería practices define a whole lifeworld, framed over a 1,000 years. Chinampería is a way of connecting with the land, with the water, the food and, most importantly, with others in the community. It is an issue of identity. The systemic dissolution of the chinampería practices is also tearing apart the social-ecological fabric that gives life meaning to those embedded in this system.

## Exploring Agency Through a T-Lab

The Transformation Laboratory project (or T-Lab) was part of an international network^6^ created to experiment with designed and facilitated processes to address complex social-ecological issues from the diverse perspectives of different groups of stakeholders (The Pathways Network, 2021). T-Labs were developed from the concept of Social Innovation Labs (Westley et al., 2015). The transformation aspect of the projects meant that the experimentation aimed to: explore the diverse framings around a challenge, find change-makers and strengthen their individual and joint capacities to more effectively address the challenge; develop change strategies that test multiple solutions which could help to solve the challenge; and create early prototypes of interventions (i.e., new forms of governance, new type of practices, etc.) to build momentum for action (Pereira et al., 2021).

The Xochimilco T-Lab in Mexico City included a group of 19 participants. Participants included local agricultural producers from different areas and types of production, inhabitants of irregular settlements on abandoned chinampas and ecologically protected land, residents, members of NGOs, academics, and government officials at the federal level. Selection criteria included: (1) diverse types of knowledge about the area; (2) actionable social networks, e.g., through previous capacity-building projects, organized collective work, institutional affiliations, etc.; (3) capacity and willingness to experiment with different approaches; (4) determination and will to both conserve social-ecological attributes of the system and change the current hindering conditions; (5) some sense of attachment to the place, i.e., Xochimilco wetland; (6) experience in alternative activities, e.g., organic farming, ecotourism, eco-technologies; (7) solidarity and empathy with respect to other group members; and (8) experience working on problems of community development and grassroots innovation (Charli-Joseph et al., 2018).

The objective of the Xochimilco T-Lab was to foster a reframing process about the problem of Xochimilco's degradation: reframing the position of participants in the social-ecological system and reframing the dominant narrative about their own agency. Over the course of 2.5 years and 12 group interactions, the project deployed two suites of methodologies. A set of methods fostered interpersonal changes among participants and intrapersonal changes in the group. These methods mapped the participants' personal and social networks, their framing of the social-ecological system using cognitive mapping technics, and Q-method to understand the value and moral aspects of the participants' views on the system (Charli-Joseph et al., 2018; Eakin et al., 2021). We designed another set of methods and techniques to promote collective agency. Both suites of methods focused on reframing the problem space to develop new opportunities for participants to connect with the social-ecological system. Although the methods and techniques involved reflexivity, they also included intense hands-on work, such as walkshops (Wickson et al., 2015), diverse forms of participatory mapping, including building group dioramas of the Xochimilco landscape and values and meanings mapping, and learning farming techniques from the chinamperos (Ruizpalacios et al., 2019; Manuel-Navarrete et al., 2021).

### Results From the T-Lab

A recurring theme in the T-labs were people's testimonies about the loss of the wetland and their culture. Our interest in agency led us to see the other side of the coin: the feeling of not being able to act; making sense of the participants' original feelings of helplessness is the subject of this article. The results of the whole project will be presented in another venue. It must be said that the group formed through the T-Lab project remains active and has identified new ways to engage and address the environmental problems that concern them as a collective.

The results of the values and meaning mapping, as well as cognitive mapping, and open interviews provided insights to our current focus on helplessness and loss (see Charli-Joseph et al., 2018). The value and meaning mapping emerged from the work of mixed groups of three or four participants who together created a diorama of the area, highlighting those attributes that were relevant for them. This activity was followed by a session to deliberate and reflect over those aspects that participants considered relevant to preserve in the system, even in the face of current environmental and social degradation. During this activity, the most valued aspects of “being from Xochimilco” were identified as their autonomy and self-sufficiency as food producers, aesthetics of the landscape, and identity. Xochimilco wetland landscape was unique and plays an important part in local imaginaries. Chinamperos have high regard for their capacity to produce food for their own and for others. Their appreciation for the aesthetics of the landscape is instrumental in their sense of place. Identity was the other aspect that was at the core of elements valued associated to Xochimilco. They see themselves as people that historically have resisted, first to the Aztecs, later to the Spanish and more recently to the Mexican government (Eakin et al., 2019).

As reported in Charli-Joseph et al. (2018) we used mental modeling techniques to identify two dominant narratives about the situation of the wetland in general. The first narrative centered on the impact of urbanization, the second one on water quality. According to these narratives the main drivers of the degradation of Xochimilco were immigration and irregular settlements, and unsustainable farming and livestock managing practices. Both drivers connected strongly with the pollution of water. Irregular settlements do not have basic services such as piped water and sewage systems, and as a result all wastewater goes to the channels. The use of fertilizers for crops and the waste produced by livestock farming also end up contaminating the channels. Other issues were reported as part of these mental models, such as chinampas abandonment, loss of traditional values, lack of participation of civil society and lack of chinampa conservation policies.

These narratives were also confirmed during interviews, and reflected the acknowledgment that underlying processes degrading the environment were having an impact on chinamperos' self-esteem, as well as the destruction of the landscape. It was not only that new houses were being built on chinampas but also that this construction implied a rejection of chinampería by the younger generations, who were not interested in spending hours under the sun, full of dirt and earning very little; they would rather have any other “urban” job. They also felt that society has a low appreciation for being a chinampero and a farmer:

➢ “*I do not introduce myself to others as chinampero but as a producer. From the outside, to be a farmer is something derogatory. That is the biggest problem with those who have never lived here [in Xochimilco] […] When they talk about a farmer or a chinampero they think this is a person who has no studies. We touch racism, or I should say discrimination.”*

As for the impact of water quality on self-esteem, one chinampero mentioned that their products were sometimes hard to sell:

➢ “*People don't purchase products from Xochimilco because they know they are contaminated with treated wastewater…it doesn't even pass through their heads that the water is treated, rather they think ‘dirty’ and from the beginning this [framing] sticks. (Eakin et al.*, *2019**)”*

Some more impactful testimonies attributed the increased number of cases of alcoholism, domestic violence, and even suicidal attempts due to chinamperos being unable to sustain their families as the land is not as good in producing as it used to be. Xochimilcas were proud food producers. The fact that this heritage is being lost is one of the hardest blows for them with an important impact on their self-esteem, in their sense of self-efficacy and, on a more basic level, on their very sense of agency. As expected, positive, although nostalgic emotions were attached to the chinampa: the interviewees would speak of their personal satisfaction, their memories of childhood, and the “feeling that one is contributing to a better world” in relation to the chinampería.

In sum, through these different methods and techniques and several group interactions we found that the participants perceived the causes of change to be external. They were unable to identify specific causes of degradation (there are too many and too intertwined). The participants conveyed a sense of being spectators as external forces degraded the social-ecosystem. Yet, they directly experience and suffer these changes. They communicated a sense of lack of control over the situation and, as reported by two of the participants, a long history of repeated failed interventions on the system aimed at restoring the ecosystem and farming practices. Finally, they perceive others as seeing them as “dirty peasants” or campesinos. This low self-esteem is associated with alcoholism, domestic violence, and other social ills. Above all, they conveyed a longing and nostalgia for a Xochimilco from the past that is long gone.

## Agency

In order to understand the effects of the erosion of Xochimilco's social-ecological system on chinampero's agency, we would like to present briefly the concept of agency we rely on. An agent^7^ is not only someone who can act and causally affect its environment; an agent is someone who also possesses a sense of being an agent, which consists of a self-construal as someone who can skillfully manage a situation. Such a sense is primarily an affective bodily felt sensation of an “I can” and it only emerges in interaction with the world. In the first part of this section, we will describe what we take to be an agent as necessarily embedded in a shared sociomaterial context.

In the 20th century, pragmatists, such as Dewey (1958) and James (1981), as well as phenomenologists, such as Maurice Merleau-Ponty, independently defended that consciousness and our bearings in the world are fundamentally defined by what we can do (“I can”), preceding what we think (“I think”) (Merleau-Ponty, 2013, p. 139; Toadvine, 2019). Following this tradition, we take the most basic engagement with the world to be action, which involves an embodied tacit know-how, and precedes any reflective and analytical attitude. The world is always one that is firstly meaningful not because of what we think of it but because of what we can or cannot do in and with it. The pragmatic understanding of consciousness as a relation of action is one of the foundations of Ecological Psychology (Heras-Escribano, 2019) which, in what follows, provides the foundation on which we build a theoretical framework to understand the relation between chinamperos and their environment.

Ecological psychology conceptualizes the “I can” connection between a subject and the world that makes meaning possible in terms of affordances. An affordance is an opportunity for action that an aspect of the environment offers to an agent (Chemero, 2011; Gibson, 2014; Rietveld and Kiverstein, 2014). Such opportunities are the result of two fundamental components, on one side there are the abilities and skills of an agent and on the other the properties of a sociomaterial environment. Affordances are relational and emerge only when these two meet^8^. From this perspective, agent's abilities and skills are part of the agent's bodily constitution—including the brain- and in this sense the body establishes the background of possibilities for action. However, the perception of affordances is a two-way road, and meaning can only emerge because some properties of the world are salient and can be perceived by an agent because of her skills and abilities to engage with it. For example, we can't engage with those aspects of objects that are only visible in infrared light simply because we do not have the visual apparatus for it, at least not without some technological support.

Yet, one may ask, where do these abilities and skills of an agent come from in the first place? Our biological bodily constitution establishes certain constraints and possibilities for the subject to act and to develop a set of skills and abilities. These abilities, however, are shaped throughout a biographical history of being part of a social and cultural group: through a variety of cultural practices, we have been taught to get involved with the world in certain ways and not in others. Most of our skills and abilities, if not all, are instantiations of the social and cultural practices of the social group(s) that we are part of.

As Rietveld and Kiverstein (2014) have argued, social practices refer to the relatively stable and regular ways of doing things shared by a community. These patterns weave together and constitute a form of life. A form of life is never exclusive to an individual but it connects a population through and through by means of shared and common ways of doing and know-how. For example, the practice of chinampería involves certain forms of farming, it requires cultivating and maintaining an ecosystem formed by the chinampa and for all this to happen, chinampería requires the participation of the whole family and its organization in particular activities, divided among its members. Chinamperos would also know how to navigate the channels, exchange their products for other goods, organize their daily work activities according to the demands of the season, etc. Moreover, the evolving set of chinampería practices and knowledge have been inherited by generations and shared among peers for hundreds of years; practices and knowledge that connect people to people, and people to place. For us, humans, a social and cultural species, a form of life implies shared practices and expectations that are maintained as we constantly enact them in situations that appear to us at every moment of our lives.

This idea of growing skills and abilities as part of a social group in a particular environment allows us to better understand what is at stake in the erosion of a community's historical environment. We posit that environmental anguish and its psychological toll cannot be reduced to the disappearance of vegetation and animals, not even of a landscape, but to the erosion of a social-ecological system that makes possible the permanence of the ways and practices of a community. It is ultimately the social-ecological system that sustains people's deepest know-how. This social-ecological system is formed by landscapes of affordances that interconnect and are relative to the available skills of a community; landscape of affordances and skills are constitutive of a form of life (Rietveld and Kiverstein, 2014; Ramstead et al., 2016).

Affordances never come alone: First, an affordance is always part of a wider landscape of affordances relative to the skills available in a community; for example, a chinampero might not know how to make the traditional mudflats used for sprouting seeds but she can recognize the tools as affordances available to others in her community to make them. Rietveld and Kiverstein (2014) call this network of affordances available to a community “a landscape of affordances”.

Second, at the individual level, affordances intertwine with each other as a spectrum of possibilities for action in which engaging with one leads the agent to follow others. For example, corn seeds may present the chinampero an opportunity to plant them, or to start a conversation with a fellow farmer; in turn, the conversation might be an opportunity to gossip or to exchange seeds which my lead to a discussion of the particularities, the nuances of planting a seed of a given variety, its benefits and its history, etc. Bruineberg and Rietveld (2014) call this network of possibilities available to an individual a field of affordances.

The idea of a field means that an agent in a particular situation—just like the chinampera in our example- will be introduced to different affordances with which she can engage simultaneously. It also implies that by engaging with a given affordance other opportunities for action are revealed to the agent. Her tacit know-how, skills and abilities make it possible for her to identify those relevant affordances and to choose the affordances relevant to behave appropriately as the situation unfolds and demands (Rietveld and Kiverstein, 2014; Ramstead et al., 2016). In short, a field of affordances refers to the agent's here-and-now possibilities of action, as well as to the open horizon of actions.

On this basis of this theoretical foundation, our concept of an agent is defined as follows: An agent is an individual that is fluent in navigating a form of life by skillfully engaging with the sociomaterial affordances of shared practices within a shared form of life.

An important consequence follows from this conception of agency: an affective experience of being in the world. Being an agent implies that the individual is directed to the world as a space with which she can engage because she has the skills to do so—van Dijk and Rietveld have expanded on this phenomenon of directedness and have called it “skilled intentionality” (van Dijk and Rietveld, 2017). This intentionality is felt as being able to fluently navigate a form of life, that is, the feeling that one can aptly move in a situation, and it results in a general *feeling-at-home* that precedes every particular emotion and motivation to act of the individual. This *feel* is bodily felt, a primordial *affective state* that connects our disposition toward a situation and the situation itself. This way of conceiving affectivity brings to the forefront the fact that it is strongly intertwined with agency, as acknowledged for a long time (Frijda, 1986; Fuchs, 2013; Fuchs and Koch, 2014; Slaby and Wüschner, 2014). This affective experience includes both one's felt sense of self in relationship to the world, that is, one's felt identity (Slaby, 2012), as well as one's feeling of belonging to a community where skills and activities interweave to maintain a form of life.

Recognizing this affective component allows us to understand, as we show in the following section, why chinamperos' agency, their affective intentionality toward the world, and their own identity are deeply affected by environmental degradation of Xochimilco's environmental degradation; and should be considered a collective problem rather than an individual matter.

## The Collective Nature and Politics of Affectivity

In this final section, we argue that anxiety, depression, hopelessness and affective unrest attributed to environmental degradation should be considered a social problem and, therefore a political matter through and through. As explained in section Xochimilco Social-Ecological Wetland, Xochimilco environmental degradation has many intertwined social, political, economic and ecological factors running in parallel, e.g., climate change, land use change, surplus of Nitrogen, and Phosphorous in aquatic and terrestrial ecosystems due to crop fertilization, conflict among farmers to access water, salinisation of the soil because channels and wells desiccation and flooding, among others (Eakin et al., 2019, p. 6–7). From the accounts of the T-Lab participants, we could infer and hypothesize that the complexity and wickedness of the environmental problems impact the affective lives of chinamperos which, in turn, may generate political vulnerability, but to confirm this, further research would be needed.

Let us elaborate further about the impact of environmental degradation on the affective lives of chinamperos. The dynamics of affective states do not change randomly, but rather depend precisely on the flow and feedback between environment and subject. Affective states can change as the result of the environment not offering the conditions for the emergence of affordances that match the subject's skills and abilities. The environment may not offer such salient aspects simply because it does not have them anymore, which is the case in environmental change and degradation. In the case of the wetlands of Xochimilco, the environmental change in the quality of soil and water, the desiccation of wells and channels and the reduced availability of water implies that the affordances for farming activities of chinamperos have disappeared as the material conditions to exercise their skills are no longer there. This could result in a sense of ineffectiveness. Then anxiety, desperation and a sense of failure can become dominant affective states, that in turn contribute to a diminished sense of agency (Bandura, 2018, 1977). Similar results have been found in adaptation to climate and environmental change literature. Research has shown that the perception of low self-efficacy is positively correlated to absence of motivation to take action toward adaptation (Grothmann and Patt, 2005, p. 203; van Valkengoed and Steg, 2019).

Moreover, according to Slaby, a person's self-awareness and self-construal in terms of agency are connected to the person's affective states that reflect their position in the world according to their sense of abilities. They are about the situation that one is confronted with—i.e., the world— defining the ways one will approach it and what one can do and how capable one is to perform in it (Slaby, 2012). As it defines one's position in the world, the sense of ability builds a sense of being situated in a particular situation, a self-construal that stands on the “what I can” and “what I cannot”. Such a position allows one to perceive some affordances in the situation and others to be unnoticed. As material affordances are no longer present, chinamperos' feel the irrelevance of their skills leading to a self-awareness and a self-construal of incapability where affordances are not seen.

As the erosion of affordances becomes more pronounced, affective states may get trapped in a downward spiral where there is a greater loss of the sense of agency and an increasing presence of anxiety and depression. The loss of agency due to environmental degradation prevents other affordances to emerge, therefore the subject cannot execute the skills and abilities proper to the form of life that she is part of. The inefficiency of skills and abilities, in turn, contributes to a feeling of disconnectedness from the world and the interactions with the world that still take place can begin to be perceived as superfluous and, in the worst scenario, meaningless. In the case of chinamperos, this scenario has been repeated over the years as environmental degradation continues to aggravate and the many workshops and interventions are perceived as insignificant, of little consequence or even counterproductive. Thus, the feeling of lack of agency, disconnectedness and meaninglessness might become a long-term affective mood which manifests as depression and anxiety. As shown by de Haan and colleagues, under a condition of depression and anxiety, fewer affordances are perceived (de Haan et al., 2013; Slaby et al., 2013; Krueger and Colombetti, 2018) which means that, even if affordances were available, agents in such affective states would not be able to perceive and engage with affordances even if they were available in the environment again.

This phenomenon might be an instance of learned helplessness, a psychological phenomenon in which individuals, after having been repeatedly exposed to stressors over which they have no control, continue to passively endure the effects of the stressors even when they regain control over it (Seligman and Maier, 1967). This might contribute, together with the external stressors as the factors we identified in Section Xochimilco Social-Ecological Wetland, to chinamperos' feel that they stand as spectators of Xochimilcos' transformation by what they perceive as external forces, they feel alienated from the social-ecological system, they feel disconnected from that world, and they cannot find any meaningful way to intervene in its fate.

From an anthropological perspective, chinampería is not just farming, instead it is one of those activities referred to as a *total social fact* (Mauss, 2002). This is to say that historically^9^, chinampería was truly a set of practices that would touch every aspect of Xochimilco's social, cultural and ecological life, even those not directly related to farming. Chinampería would organize the household, it had a great role in the economy and politics of the community (Canabal Cristiani, 1997). Chinampería would also organize the landscape and would directly be incorporated into the biophysical dynamics of the system.

The recent history of Xochimilco offers many examples that show the interconnectedness of chinampería with the life of a community and how these links have been breaking due to environmental change. Such transformations have brought conflict and new forms of social organization (Eakin et al., 2019, p. 7). Its consequences reach technical, social and cultural aspects of chinampería as chinamperos need to use different methods for farming, they need to hire and rent technologies that were unnecessary years ago, and in the process, they lose their highly valued autonomy and self-sufficiency supported by a complexly integrated farming system.

These changes signify a change in the form of life shared by chinamperos and therefore, a change in their landscape of affordances. As we showed in the previous section, a landscape of affordances is the environment a community inhabits that allows them to exercise the skills available in their shared form of life. A landscape of affordances is thus relative to a form of life. Mutuality skills and activities interweave to maintain the shared life of the community, as, for example, it was the case in the collective ability of chinamperos to produce food for themselves and to provide Mexico City. Thus, an individual ability to engage with affordances, for example, to prepare the mudflats for sprouting seeds, is part of a larger network of abilities in a community, such as the ability to excavate soil from the lake bed, to manually navigate punts through narrow channels, to transport produce to other parts of the city, etc., and it contributes to the maintenance of such life form. Thus, under the kind of analysis we are attempting here, it should be acknowledged that the impact of environmental degradation is not only on a specific way of livelihood but on how a whole community organizes innumerable aspects of their lives, that is how it conforms a form of life as described above.

The affective conditions just referred to, solastalgia included, are individually felt, and even when acknowledged as something that can happen to whole groups, they are typically considered to be limited to the domain of the individual. However, when dealing with environmental degradation, such affections are intrinsically about interactions with others and with the ecological environment, hence they cannot and should not be confined to a problem of the individual. The reason is that such affective conditions are grounded in the erosion of a form of life, that, in other words, result from the destruction of the socio-material or social-ecological fabric that binds people together and people and nature. This means that these affective conditions are a concern of the collective.

### Affective Conditions Are of Political Concern and Generate Political Vulnerability

In this last section, we want to insist that the affective dimension of chinamperos needs to take a political spin. Our rationale is that affectivity is crucial for agency and agency is required for social-ecological transformation and a community's continuous search for wellbeing.

Affectivity is relational, and we follow Spinoza in that it is political. For Spinoza, affect refers to the interaction between bodies—human and non-human—through which their capacities or powers are enhanced or diminished. In this sense, individuals realize their power only in contact with others, or as being part of an “ecosocial matrix of other bodies, affecting them and being affected by them” (Protevi, 2009; Slaby and Bens, 2019, p. 342). Moreover, the political aspect of affectivity is about freedom, not so much as in individual liberties but as a matter of the collective. Aurelia Armstrong has rightly pointed out that freedom or autonomy in Spinoza can only take place as a social process that involves effective interactions in a community for the realization of those who are part of it (Armstrong, 2009). Freedom can be attained only in the context of the dynamics of the ecosocial matrix we just referred to Slaby and Bens (2019).

In the light of Armstrong's reading of Spinoza's work, our insight into the Xochimilco situation is that environmental degradation is preventing the appropriate dynamics that would lead to the realization of freedom and autonomy of its community. In other words, as the environment erodes, a form of life that has endured for centuries has been breaking and tearing apart, severely reducing its members' agential capacities and leaving them in a situation of an existential void that causes affective stress. As we see it, this situation demands a political consideration that can be framed as a problem of social and environmental justice. The possibilities of a community to act and pursue its wellbeing are severely limited as the affordances due to environmental degradation are reduced. In other words, the fabric that allowed the community to strive in Spinozist terms is coming undone.

Finally, a community with a severely damaged sense of agency cannot properly act, however, we also consider the possibility that a community for which freedoms have been reduced can become politically vulnerable. For example, Galindo Marquina (2017, 2020) has reported on the role of clientelism in relationship with the agroindustry, in which there is an exchange of political support and loyalty from chinamperos for accessing resources offered by governmental programs. There may be different reasons for someone to engage in clientelist practices, however, clientelist networks can become spaces where they can gain access to the resources they need to keep farming. However, we do not know if participating by in these networks, chinamperos also find, at least partially, the support that was formerly given by a community united by a shared form of life. This is an open question that requires further empirical research. The idea, nonetheless, is that this support can come from political groups that do not necessarily portent a sustainable, just or legitimate cause. As the environment degrades and the community loses, someone else directly or indirectly wins, be it political parties, the agroindustry, or the construction industry that uses the wetland as their debris wasteland. Examples of situations like this abound.

## Conclusions

For our Transformation Lab, agency was the central topic. It is the expression of human concern and drives toward the wellbeing of the self and that of our community. As a matter of concern, agency is inextricably linked to our affective life. In a situation in which the conditions for people's know-how and practices are shattered, their sense of agency begins to go weary to the point where people experience powerlessness and hopelessness. As Fisher (2009) and many others have pointed out, the world system, with its dominant social, political and economic system based on the logic of perpetual growth, creates conditions that limit and channel people's agency. Environmental degradation is one of these forms of limiting agency (Barnett et al., 2016; Maiese, 2022).

In this context, climate change inevitably brings with it environmental changes that represent the degradation of affordances and a negative impact on the form of life of populations. According to our argument, this situation creates a sense of inefficiency of shared know-how, loss of control, and therefore a sense of limited and thin agency. Such impacts on the life of people and communities may pass unseen, mostly due to research on climate change primarily being focused on its biophysical and economical aspects. Nonetheless, the impact on culture and affective life of populations is highly meaningful in the context of adaptation to climate change, especially for those populations whose livelihoods are heavily resource-dependent (Adger et al., 2013; Tschakert et al., 2013; Brown et al., 2019; Marshall et al., 2019). The risk of an increasingly weakened sense of agency is that it might create a vicious cycle where affective stress caused by environmental degradation limits and biases the possibility to see opportunities for action (Yang et al., 2018), linked to conditions such as depression, hopelessness, and inaction.

We believe that our work offers a theoretical approach that can contribute to this discussion about adaptation to climate change. We are convinced that Xochimilco is not an exception, but that there are many similar cases over the world, especially in the Global South (Marshall et al., 2007; Poma, 2018; Cooper et al., 2019; Iniguez-Gallardo et al., 2021). In this work, we have shown that agency and the sense of agency in people are completely related to affectivity. Agency takes form in the context of a shared set of know-how and practices that constitute a form of life and that are executed in particular situations. Such a form of life has evolved along with a sociomaterial environment. When the sociomaterial environment changes in directions that make know-how and practices inefficient, the world begins to appear chaotic, their lack of effectiveness affects people's agency and their corresponding sense of agency. Further research is needed to better understand the relationship between environmental degradation such as observed in Xochimilco, and moods of hopelessness, depression, and anxiety. Moreover, we posit that enactivism and ecological psychology can contribute substantially addressing this topic. Finally, we have argued that the loss of agency and the affective conditions that accompany this loss should be a matter of political concern, given that an agentially depressed community is not in the position of engaging in action toward procuring individual and community wellbeing. Further, a depressed community might be politically vulnerable and subject to political abuse.

## Data Availability Statement

Publicly available datasets were analyzed in this study. This data can be found here: https://github.com/sostenibilidad-unam/tlabs.

## Ethics Statement

The studies involving human participants were reviewed and approved by Arizona State University IRB. The patients/participants provided their written informed consent to participate in this study.

## Author Contributions

JS-G and LM conceived the work. JS-G, DM-N, HE, and LC-J designed the work. PP-B and BR contributed with data search and identification. JS-G, DM-N, HE, LM, and LC-J drafted the work and revised it critically for important intellectual content. All authors contributed to manuscript revision, read, and approved the submitted version.

## Funding

Universidad Nacional Autónoma de México, PAPIIT IT300220 supported the research about the relationship between agency, the environment and wellbeing.

## Conflict of Interest

The authors declare that the research was conducted in the absence of any commercial or financial relationships that could be construed as a potential conflict of interest.

## Publisher's Note

All claims expressed in this article are solely those of the authors and do not necessarily represent those of their affiliated organizations, or those of the publisher, the editors and the reviewers. Any product that may be evaluated in this article, or claim that may be made by its manufacturer, is not guaranteed or endorsed by the publisher.

## Footnotes

^1^A standard definition of a social-ecological system can be found in Redman et al. (2004) “1. a coherent system of biophysical and social factors that regularly interact in a resilient, sustained manner; 2. a system that is defined at several spatial, temporal, and organizational scales, which may be hierarchically linked; 3. a set of critical resources (natural, socioeco- nomic, and cultural) whose flow and use is regulated by a combination of ecological and social systems; and 4. a perpetually dynamic, complex system with continuous adaptation”.

^2^Ramsar Convention is an international treaty for the conservation and sustainable use of wetlands over the world. Specific sites are registered as Ramsar sites. That is the case of Xochimilco. https://rsis.ramsar.org/es/ris/1363.

^3^Xochimilco UNESCO World Heritage site: https://whc.unesco.org/en/list/412.

^4^This information was shared by Patricia Pérez Belmont as part of her own PhD research.

^5^Minimum wage for Mexico City is $172.87 Mexican pesos a day or approximately USD 8.06 dollars. Farmers that profit USD 300 ($6432 Mexican pesos) would surpass the monthly minimum wage of $5186 Mexican pesos.

^6^The Pathways Network included hubs from the UK and Argentina, China and Kenya, and India and North America (Mexico-US).

^7^From an enactivist perspective any living thing counts as an agent, from a New Materialism and Latourian points of view, agency may also be defined and not limited to humans or living things, however, for this manuscript we are strictly concerned with human agents.

^8^We appreciate one of the reviewers noticing that different scholars may have different understandings of the notion of affordances. Although we see them as relational, authors such as Turvey (1992), Reed (1996), and Stoffregen (2000) among others, conceive affordances as properties of the environment, either as physical properties or playing role in natural selection. For modern reviews about the ontology of affordances see Chemero (2011), Heras-Escribano (2019), and Kiverstein (2020).

^9^Contemporary Xochimilco is socially, culturally, politically and economically very diverse and by no means its population can be homogenized under a single label such as the “people of Xochimilco”. However, for a long time, since its foundation a 1,000 years ago, it remained as a small agricultural community where farming was a central aspect of life (Canabal Cristiani, 1997).
